# The lived experience of female genital cutting (FGC) in Somali-Canadian women’s daily lives

**DOI:** 10.1371/journal.pone.0206886

**Published:** 2018-11-06

**Authors:** Danielle Jacobson, Emily Glazer, Robin Mason, Deanna Duplessis, Kimberly Blom, Janice Du Mont, Navmeet Jassal, Gillian Einstein

**Affiliations:** 1 Dalla Lana School of Public Health, University of Toronto, Toronto, Canada; 2 Institute of Medical Sciences, University of Toronto, Toronto, Canada; 3 Women’s College Research Institute, Women’s College Hospital, Toronto, Canada; 4 Department of Psychology, University of Toronto, Toronto, Canada; 5 Department of Family & Community Medicine, University of Toronto, Toronto, Canada; Migration Institute of Finland, FINLAND

## Abstract

Many of the Somali women who have immigrated to other countries, including Canada, have experienced Female Genital Circumcision/ Mutilation/ Cutting (FGC). While there is literature on the medical aspects of FGC, we were interested in understanding the daily life experiences and bodily sensations of Somali-Canadian women in the context of FGC. Fourteen women living in the Greater Toronto Area were interviewed. Interview data were analyzed using a phenomenological approach. We found that the memory of the ceremonial cutting was vivid but was frequently described with acceptance and resignation–as something that just is; that was normal given the particular context, familial and cultural, and their young age. Most of the women recounted experiencing pain and discomfort throughout their adult lives but were intent on not noticing or giving the pain any power; they considered themselves healthy. The following themes emerged from our interviews: *Every Body Had It*: *Discussing FGC*, *I’m Normal Aren’t I*?, *and Feeling in My Body–*all themes that work at normalizing their bodies in a society that they know views them as different. They dealt with both pain and pleasure in the context of their busy lives suggesting resilience in spite of the day-to-day difficulties of daily life.

## Introduction

Female Genital Circumcision/ Mutilation/ Cutting (FGC) is a cultural practice that includes the cutting and/or removal of portions of the female genitalia [1]. FGC is practiced across 30 African countries, Asia, the Middle East [2], and in India and Sri Lanka by the Dawoodi Bohra [3]. Type IIIb FGC is the most extensive type of genital cutting and the most frequently practiced in Somalia [1]. In Type IIIb, the external clitoris is excised, the labia minora cut, and the labia majora sutured together (infibulation). This leaves a small introitus for the passage of menstrual blood and urine [1]. In Somalia, a woman with FGC is not only more marriageable, but is also recognized as beautiful, clean, pure and feminine [4, 5]. As of 2008 it is estimated that 97% of Somali women, aged 15–49 have FGC [1].

In 1988, Siad Barre’s regime attacked Northern Somalia and civil war broke out. In 1991, the government collapsed. Through this period, many Somalis began immigrating to Western countries [4] including those of Europe, North America, Australia and New Zealand [2]. Toronto is now home to over 55,000 Somali immigrants [6] with some studies estimating that there are 70,000 to 100,000 Somali immigrants in the greater Toronto area (GTA) comprising the largest group of African immigrants in Canada [7]. Having left their homes, many now face racism [8] and being visibly different in a new cultural context [4]. This extends and impacts across the full spectrum of daily life. In addition, as with many immigrant groups, Somali women with Type IIIb FGC experience adverse biomedical [9] and social complications that impact their everyday lives [10].

We wanted to understand Somali-Canadian women’s experiences of FGC and how they experienced their bodies in their current, Canadian lives. In particular, East African immigrant women with FGC, specifically Somalis, who are most concentrated in Europe, the United States and Canada [11], are viewed through the lens of how their FGC has affected their reproductive health [12, 13, 14, 15, 16]. We wanted to extend the conversation about health beyond the common emphasis on reproduction to include other issues and infrequently addressed needs [17]. We were particularly interested in Somali-Canadian women’s experiences of pain and pleasure. Other reports have discussed pain related to the loss of country [4, 18] and the immigration context [19]. We wanted to understand Somali-Canadian women’s everyday whole body experiences and the connection between FGC, and women’s lived lives. In so doing, we hoped to contribute to a better understanding of their lived lives and health needs in their new home country.

## Research methods

The qualitative interviews reported here were part of a larger study to understand pain in women with FGC. The larger study was designed in two parts: a qualitative portion to understand the women’s FGC experiences and how they might influence their lived lives including, bodily sensation (reported here), and a second part focusing on quantitative pain information (reported separately). Here we report on just the qualitative portion which did not focus on pain, but instead on lived experience, although included questions about pain and pleasure. The design, implementation, data collection, and analysis all include the consolidated criteria for reporting qualitative research (COREQ) with most of the items on the checklist considered [20]. Ethics approval for the study was obtained from the University of Toronto Research Ethics Board.

### Theoretical framework

Interpretive phenomenology framed our methods of data collection, coding and analysis [21]. Phenomenology focuses on understanding three aspects of embodiment: (a) the nature of experience from the perspectives of people experiencing a phenomenon; (b) the essence of and commonalities among people’s experiences and; (c) the ways in which people experience the world through their bodies. It strives to build a whole picture of an experience, including bodily/ sensory experiences [22] and was therefore highly consistent with our study interests. Data were collected via in-depth interviews with a purposive sample of 14 community women with FGC. The sample size was based on the recommendations in the literature that phenomenology studies should have small and purposeful samples in order to gather rich and in-depth data [22].

### Community advisory group

It was our goal for the research to be meaningful to the women we interviewed. Thus, we established a community advisory group (CAG) to provide input on our underlying question as well as to provide advice and input on all the instruments used. The CAG supported the study objectives and thought them important [23]. Our CAG was comprised of three members of the GTA Somali community working at Community Health Centers. The CAG provided consultation about the value and direction of the research project, as well as assistance with translational issues (cultural and linguistic), selecting appropriate research instruments and participant recruitment. Over the course of the study, the CAG and the research team met approximately once every three months.

### Participant sample and recruitment

We chose to study Somali-Canadian women because FGC is carried out relatively late in their lives (ages 4–15) [24] and most women have Type IIIb, a type which involves the most significant cutting of nerve and muscle [25]. Fourteen women were recruited by purposive sampling from the GTA Somali community. The CAG recruited Somali women of reproductive age (approximately ages 18–45) who had undergone Type IIIb FGC, had no chronic illnesses, and had lived a minimum of two years in Canada. Participants were told that the study was about pain (the focus of the larger study), however, they were explicitly informed that we were not interested in their genitalia. Interviews took place at a Community Health Centre.

Informed consent was obtained at the beginning of the interview. Most of the participants had at least a high school education and were literate in English. However, consent documents were printed in both English and Somali and a member of the CAG was present to help translate and interpret. Participants could ask questions or comment in either English or Somali before the interview began and when literacy was an issue, provide either verbal or written consent. Participants had the option of completing just the first part of the study (the interview portion) or continuing on to complete the second part which involved a physical assessment of genital sensitivity. Participants received monetary compensation for their time and expenses.

### Data collection procedures

Semi-structured interviews were conducted by RM (PhD) and DD (MPH). Interviews lasted an average of 1.5 hours. All interviews were carried out in a private room in a Community Health Center. The interviewer introduced herself, offered the participant fruit and tea, and explained the study and obtained consent. The participant was asked in advance if they would like to have an interpreter present and if they said, ‘yes’, one of the CAG members attended to translate.

Key questions included:

1. Tell us your place of birth, family, and your immigration to Canada
2. Tell us your FGC story
3. Take me through a day in your life and how your body feels
4. When does your body feel best/worst
5. Five words to describe FGC and/or how your body feels

We analyzed each interview for themes and interviews were conducted until no new themes arose. Field notes were taken and consulted. All interviews were transcribed into text. The two interviews conducted in Somali were translated and transcribed by a Somali language expert who had signed a confidentiality agreement. Transcriptions were cross referenced with the original audio interview by a member of the research team not involved in the interview. Any discrepancies in wording were discussed and consensus reached. If there were still outstanding issues regarding meaning, the transcription was brought to the CAG for their input. To ensure confidentiality, transcripts were anonymized and identifiers were kept separate from the transcripts. All data were kept on an encrypted computer or in locked file cabinets in the primary investigator’s locked office.

We recognized that we were not members of the Somali community but Caucasian academics. The principal researchers reflected on their unique social locations including their positionality within academia: graduate student in public health (DJ, DD), researcher on intimate partner violence (RM), student researcher interested in pain and gender (EG), and neuroscientist interested in the neurobiological repercussions of FGC (GE). Positionality was talked about often during the carrying out of the study and the determination of themes—power relations between the researchers and the participants, varying religions, and professions—since unspoken qualities of researchers may have an impact on the data generated and analysis [26, 27].

The researchers consulted the CAG comprised of Somali women with FGC. We consulted them to glean cultural nuances associated with the Somali-Canadian community. The CAG helped provide insight on matters that the researchers felt they could better understand with consultation and cultural expertise. With respect to reflexivity within researcher assumptions, before entering the research, we were aware of reports of women with FGC, after immigrating to Western countries, feeling a sense of remorse and loss because of their circumcision [18, 28]. Thus, we expected to hear about stressful experiences, or pick up on melancholic tones during the interviews because we were sympathetic to the difficulties associated with FGC and immigration.

### Data analysis

All but two interviews were tape recorded and later transcribed. Hand written notes were made on two unrecorded interviews. All personal identifying information was removed and numbers assigned to ensure anonymity. In this paper, names common to Somali culture have been assigned to each participant.

Analysis followed steps adopted from Sadala and Adorno [29] who used interpretive procedures informed by Merleau-Ponty [30]. Transcripts were reviewed for correct transcription by at least 2 team members before uploading into NVivo (version 9; QSR Software International). Once uploaded, in order to obtain a view of sensitizing concepts, transcripts were re-read independently and multiple times by four readers who compared notes [31]. Transcripts were coded into units of significance. Ongoing reflection and analysis of transcriptions and codes led to the development of themes and subthemes by the research team [32].

Upon completion of the study we invited all participants to a presentation of the themes and findings understanding that other participants would be there and thus, their participation, though not their individual information, would no longer be anonymous. Only 3 attended at which time they provided feedback that was incorporated.

## Results

### Participant demographics

The average age of the participants was 38, ranging from 21–46. The modal age of FGC was seven years old but ranged from 6 to 13; three women could not recall the age when they were cut, so an average for the group was not obtained. Six participants reported having FGC under local anesthetic, four had no anesthetic and four could not remember. All participants reported they had Pharaonic circumcision (which we assumed to be Type IIIb) and were completely or partially de-infibulated for marriage/sexual intercourse and/or childbirth. The average time in Canada was 13.9 years, with a range of 2 to 21 years. Eleven of the 14 participants had lived in Canada since the early 1990s. Most of the women were married and one was divorced. Five had completed high school and six, college or university. Most were employed in daycare or education. All had at least one child; six had delivered their children by C-section and six by vaginal delivery. Two had a mix of C-section and vaginal deliveries. The rate of C-section was high due to Toronto doctors’ unfamiliarity with infibulation. However, it is not impossible that women in Toronto could have delivered vaginally since there was one well-known (and much loved) Toronto obstetrician who did this for the community. Information about where they delivered their children was not obtained.

### Themes

Women shared their FGC stories without hesitation, seemingly eager to share their histories. The interviews elicited vivid narratives about the FGC experience. This may be a characteristic of the members of the Toronto community who participated in our study, and not necessarily of the immigrant Somali community as a whole, given the heterogeneity within this cultural group. It is also possible that our affiliation with our CAG, and focus on the whole body beyond the genitals, put the women at ease, making them willing to share their experience. The other prompts led to descriptions of their busy lives as adult women in Canada with commitments to families, children, jobs, weekend errands and relaxation, religious practice and other activities. Emerging from the women’s narratives about their FGC and their lived bodies were the following 3 themes: *Every Body Had It*: *Discussing FGC*, *I’m Normal Aren’t I*?, *and Feeling in My Body*. Although they are distinguished, the themes overlap and interrelate. Themes and subthemes are described below.

### Theme 1: Every body had it: Discussing FGC

Women tended to discuss their FGC story in terms of social pressure, comparison to others, the desire to belong in their community, and timing. In telling their stories, non-verbal expressions and postures added emotions and meaning to the words spoken. Laughter was not uncommon across the sharing of their FGC experience and interactions with family around having FGC. While they, themselves, were not silent, the women noted a silence in their community about FGC—what it was, what would happen to them, any sharing of the experience after, and the role of their fathers.

#### Something you have to pass

Many of the women interviewed described FGC using the word “natural” referring to it as something that needed to happen. Khadiija said,

"So back home it was like that…like something you have to pass…"

“Something you have to pass” conveys time and timing as well as resignation. There was an acknowledged, right time. Indeed, our participants reported their mothers were responsible for arranging their FGC and determining when it was the right time. Mothers have been discussed as both responsible for, and gatekeepers to, FGC, determining the timing for this ‘passing’ [33]. Women told us about arguing with their mothers on the timing since some of them wanted the FGC before their mothers felt they were ready. Jawaahir, reported having to plead with her mother to be circumcised. Similarly, Xaawo recalled that it was her grandmother who decided that “it was time” to have FGC. Sahra recalled her mother deciding that she must wait to undergo FGC because she was too “skinny”. Waris recalled having to wait because her mother said she was too young. Participants looked forward to the time they would have FGC. Jawaahir recalled feeling jealous seeing her peers “with their legs tied”, while recovering from FGC.

Other studies have reported the social pressure to have FGC in their respective communities including those in Somalia [34]. Not having FGC can lead to unfavorable consequences including the women being less marriageable and also being ostracized within their respective community [35, 36]. In our study, Faduma recalled an adult woman being poorly treated and talked about negatively in the community because she did not have FGC. Sahra remembered wanting to have FGC but because it was delayed due to her health, she was ostracized by her peers. She felt shame and isolation. Jawaahir also recalled the intense bullying of girls who had not gone through FGC. Because of this, she lied about her own genital status before having FGC to avoid being an outcast.

The women in our study explained that the shame of daughters not having FGC was also experienced by parents. Fawzia discussed that it is embarrassing for parents if their daughters do not have FGC. The stigma associated with being uncircumcised in a community where FGC was the norm, underscores the importance of FGC in order to belong to the community. Because FGC was such a taken for granted part of Somali culture and making a normal body, participants described that FGC “just was”, sensing that they must pass through it.

Overall, FGC was encapsulated with "what’s done is done," "just is", and "it is what it is." Participants acknowledged that FGC was the most painful experience they had. It was also acknowledged as a gateway to womanhood. Thus, the term, “something you have to pass,” conveys the normalcy of it, the importance of time in the event, and the mix of emotions that seem inevitable when one eagerly awaits and needs to have a procedure that, in retrospect, is so painful.

#### Laughter

In spite of the fact that laughter often followed a painful story, the women who participated in this study did *not* exude a somber tone. Instead, laughter and irony conveyed an overwhelming sense of a vivacious group of women.

Laughter was used in various ways. One way was used to denote power and a knowing sense of irony; it often came after women described resistance. Faduma laughed when she recalled how she accompanied her older cousin to a hospital for her FGC. When her grandmother suggested that she, Faduma, have FGC along with her cousin, she laughed as she recalled running away, realizing that she too would one day go through it. Faduma shared another story where she laughed, recalling her resistance to following the rules. She said,

“…they don’t want that [*moving your legs*] until it’s…healed…And I remember I was just moving. I was doing whatever…And my sister would say ‘Don’t do that!’ and I used to say ‘I don’t care!’(laughs).”

Laughter also characterized the sharing of intimate experiences surrounding the procedure of FGC itself. For example, Sahra told us she was not aware of how painful the procedure would be and laughed when recalling her experience. In describing her FGC experience, Canab said:

“[*I felt*] happy! I was a child. I knew nothing. I thought it was a nice thing (laughs)”.

Leylo laughed when she recalled going through FGC without feeling pain due to receiving an anesthetic.

Laugher also ran throughout the interviews when participants looked back on events that were hard to believe happened, or where they behaved in a way that deviated from the norm.

Sahra laughed when she discussed how intently she followed hygiene protocols outlined by her mother, but still got recurring infections. Although it is possible the participants may have laughed to disguise feeling awkward or in response to discomfort [37], another possibility is that they genuinely saw irony and humour in the situations they described.

Laughter occurred throughout the interviews transcending their FGC story. Women’s descriptions of their experience of becoming accustomed to differing cultural norms in Canada elicited laughter. Faduma laughed when she shared how in Somalia she was not allowed to get phone calls or answer the phone, but after immigrating to Canada, she was allowed to answer the phone. She laughed, recalling this memory of being shocked at the cultural change.

“I have to come to a new place as a…young girl I had to experience a lot. At the same time, I have experienced a lot of culture shock (laughs).”

Women also laughed when talking about sexuality and childbirth. Faduma was asked about areas of her body that she found sensitive during sexual acts, and she laughed when she responded, “Yeah in your neck area. Your ears…Breasts (laughs).” Khadiija recalled how she was very emotional after childbirth; she was very worried and cried constantly. She laughed when she described calling in the nurse when she heard her newborn crying, only to discover it was not her baby.

It is possible that our participants used laughter as a coping mechanism [37]. While laughter has not been mentioned in the FGC literature, laughter, in general, has been discussed as a coping mechanism in response to discomfort [37]. It is possible that the participants felt awkward during the interview and laughed to disguise this. It is also possible that since these were all intimate experiences under discussion, participants used laughter as an invitation to intimate conversation surrounding their FGC stories. When an interviewer laughs, it has been described as inviting the interviewee to join in and share [37, 38]. Our interviewer, DD, did indeed laugh both when she was speaking and in response to participant laughter. Her laughter may have invited the participants into intimate conversation and encouraged their own laughter surrounding their experiences. The laughter seemed to create a sense of warmth and engagement on a person-to-person level, facilitating comfort in sharing intimate details of the participants past experiences and current lives. In this interpretation, the laughter conveyed the women as people of warmth, who wanted to share.

#### Silence

We looked for silences in conversation but found few. While it is common practice to document conversational silences it has been suggested they may not hold any additional meaning [39]. However, while our participants were rarely silent, silence took on other forms. The silences of significance within this study were about FGC, itself, marriage nights and childbirth as well as father’s role in decision making and the ceremony itself.

There was silence within the community about FGC; participants told us they had never shared their FGC stories before and, if they had, it was only with their doctor. Other studies, have similarly described that FGC generally is not openly discussed within communities where it is practiced [40]. More specifically, Eritrean, Sudanese, Somalian and Ethiopian women living in the UK report that discussing FGC in the community is embarrassing [28]. Many women in our study discussed the resistance or hesitancy of speaking about FGC collectively. Hani had never talked about her FGC experience and struggled to find the English and even Somali language to capture it. Silence dwelled in the language barriers experienced when the participants described their FGC; Jawaahir and Khadiija referred to their infibulations as "that thing", or "that thing we have". It has been noted, that the practice of FGC is generally "muted" both in interpersonal discourse and cultural expressions (like poetry), despite its significance [18]. This suggests an acceptance and resignation about the practice of FGC.

Ayaan had never told her story before, and never had anyone ask her how she felt about it. She explained that in Somalia it was embarrassing to talk about and that her mother would silence her. Many women also described silences within their community surrounding the pain experienced during and after the procedure. Khadiija felt that no one talked about the pain of FGC before she had it done. However, some did know; Leylo knew from older girls that it would hurt and involve heavy bleeding.

Women also noted that the details of childbirth were not openly discussed in the Somali community, especially surrounding procedures such as caesarian section or complications surrounding childbirth. Faduma described how she experienced complications after the birth of her child and felt hesitant to discuss it with family and friends. She said,

“I didn’t ask her [*her sister*] because I didn’t [*think*] it was something [*that*] need[*ed*] to be talked about…And she never tell me anything about it…Like if I try to ask women? … They don’t want to talk about it…Cause it’s kind of a stigma, you know?”

On the other hand, participants spoke of a community silence in the context of not being told what would happen during FGC and in rumors about what might happen if vaginal tissue were not removed. Hani expressed frustration that no one spoke to her about what would happen on the wedding night, despite her effort to ask questions. She described rumours surrounding the wedding night, with no one to discuss them with:

“Some people are saying that on your wedding night your husband will take a big knife and cut into you. Nobody will really talk about it and tell you what your marriage night is like. I was trying to ask questions but nobody would talk to me…”

Even though fathers of daughters with FGC did speak their mind about their opinion on their daughter having FGC, silence surrounded their role with respect to the actual decision. Somali fathers have been reported as being responsible for sons (and not daughters), lacking involvement in their daughters’ FGC [5]. Women report that they had FGC against their father’s wishes [19]. Our participants reported that their fathers and uncles were away or disagreed with their spouses about their daughters’ FGC. Faduma described how her father was away on business in Europe when she had FGC and that he was upset when he found out that she had FGC:

“[*My father*] didn’t want us to have the circumcision…he was so mad! …When he heard… he was yelling at my mom ‘I told you not to touch my girls! I told you not to do that to my girls!’”

Khadiija and Hani’s fathers also did not agree to them going through FGC but were away during the procedure and were upset with their mothers for having it done.

One interpretation is that these accounts are a way of underscoring the mother’s important role and power in this tradition [19]. The literature maintains that men often play a passive role in this female perpetuated practice [41], despite the critical role of FGC to a woman’s marriageability [19]. When we consulted the CAG about whether or not fathers wanted their daughters to have FGC, they informed us that, of course, fathers wanted their daughters to have FGC; they would not be marriageable otherwise. Indeed, the fathers themselves would typically not marry a woman who did not have FGC. In some cases, Somali participants have said that their fathers were involved in their FGC [42] and men who are not necessarily in favour of FGC have been reported in previous literature to perpetuate the practice because of their social obligations and a desire to not disappoint their wives and mothers [43].

Thus, the fathers’ role in the practice of FGC is not always clear. For example, in Nigeria, whether or not a daughter goes through FGC is dependent on the father’s approval and not the mother’s [44]. Some literature reports that, Somali and Sudanese fathers are responsible for the purity of their daughters through infibulation, and the husband of a woman who is not infibulated is deemed untrustworthy [45] while other literature suggests Somali fathers have less say in their daughters’ FGC than mothers do [19]. It is clear that across countries and even within similar cultures there are different roles fathers play in their daughter’s FGC, which cannot be reduced to one clear cut explanation. Thus, fathers likely have a more silent role communicating indirectly about FGC [43]. With the story that the fathers are absent but with their real role in ensuring their daughter’s marriage which depends on FGC, the fathers become silent partners in the practice.

### Theme 2: I’m normal, aren’t I?

Participants described how FGC was such a natural part of the community’s culture and religion in Somalia that there was no choice of whether or not to have it. In this sense, FGC was so normal, it was embedded into the fabric of everyday life. In the context of asking about their bodies and how they felt, many of the participants talked about FGC in the context of it making a normal body. While the women discussed how FGC made them normal in Somalia, they also discussed how their FGC made them different in Toronto. The women, surprised to find that FGC is not the norm in Toronto, discussed experiences that made them feel “othered” and different from other Torontonians in the healthcare system. The women often compared themselves to other Somali’s as well as other Torontonians, especially by discussing healthcare experiences and debating FGC as a part of religious practice, wondering what normal was, and if they were normal.

#### Normal in Somalia

Just as being asked to tell us about their FGC story led the women to tell us about how FGC just was—or was normal—being asked how their bodies felt led to the women reflecting explicitly on whether *they* were normal. In this context, and carrying forward from theme one, participants discussed how, in Somalia, the act of FGC was normal and felt their bodies were *made* normal and feminine by having FGC. Leylo described how going through FGC was expected and thus not a major decision. For Aamiina, embracing and accepting the culture (including FGC) was a normal part of being Somali:

"…we don’t have [*a*] choice. We have our country, our culture…we have to accept it."

The women described FGC as a part of life and as a “turn” all girls take.

Women without FGC were considered abnormal [46] and not properly gendered [47]. When Xaawo was taught about FGC, she was told that it was an important part of maturing and getting married. Being viewed as abnormal would lead to a lack of marriageability for the women, who viewed being married as an important part of normal life.

The use of the word ‘normal’ arose repeatedly to describe the procedure, itself, menstruation, sexuality and their bodies. Faduma described nuances within normalcy in Somalia by highlighting that what is normal may differ depending on the area in which one lives. Her FGC took place at “a normal hospital” in Somalia, and she described different norms for individuals going through FGC in rural versus urban areas. She recalled that her cousin in a “normal” rural area experienced more traditional methods for her FGC, since there was less access to anesthetic and antibiotics. Leylo, who received anesthetic for her FGC recalled that she was lucky to experience the norms of city life, since girls from rural areas normally did not receive anesthetic. Leylo also used the word normal to refer to the desire to go through FGC to be like the rest of her peers. Aamiina also described her FGC experience as typical, or normative of Somali culture, including both the experience of happiness and pain.

In regards to sexuality, Khadiija described normalcy in her feelings of sexuality: “Sexuality is something that…you feel normal”. With respect to menstrual cramps, Khadiija used the word normal to describe that all women experience them, circumcised or not. The women in our study often described that they felt they were like all women in many respects other than FGC and thus, normal.

#### Realizing that in Canada they are not normal

Our participants told us they were surprised upon learning at their immigration to Canada that not all women have FGC, that it is not the norm globally, and questioned the practice which made them feel different in Toronto. This often led to women talking about the experience of being in the doctors’ office since it was in this context they were often led to feel abnormal. Sahra remembered being told she was not normal during a doctor’s visit. She related that her doctor said:

“This is your ass. What is this? Where is your vagina? …Only one hole is over there. Did you get cancer? Did they remove something?” Sahra said, “No I didn’t get cancer this is traditional.” To which the doctor…“touch[ed] his head and… said, ‘I can’t believe it.’”

Leylo recalled a similar experience when her doctor viewed her vulva during a physical examination and exclaimed, “What happened to you?!”, shocked when she explained that she had FGC. Similar to other reports that East African and Somali women with FGC often have dissatisfying health care experiences including lack of or inappropriate patient-provider communication [13], and undesirable outcomes such as increased incidence of episiotomy and severe perineal trauma compared to women without FGC [14], our participants recalled moments at doctor’s appointments and during childbirth that made them feel different, and not normal.

These negative experiences and othering may result not only from a lack of training specific to FGC [48, 49, 50, 51] but also from health care practitioners not knowing what to do when faced with a patient with FGC [52, 53]. This suggests a need for training vis-à-vis treatment of reproductive healthcare for women with FGC and a health care system that does not make women with FGC feel abnormal. It is interesting to note that how long the participants had lived in Canada did not make a difference to the experiences they reported. Women living in Canada from 3 years reported negative health experiences as did those living in Canada 18 years.

#### Comparing oneself to others

Describing healthcare experiences led the women to further contemplate if they were like everyone else. Although participants did not compare Somali birthing experiences to ones in Toronto, they did compare their birthing experiences to what they perceived to be the experiences of women without FGC, further intensifying their experience of not feeling normal. Hani recalled feeling angry, and treated differently than normal, after going through a c-section birth. She felt that she was not given enough information and did not have the same choice a Canadian-born woman would have. She described feeling pressured to undergo a c-section when she did not want to and felt that women without FGC, especially Canadian born women, would have had “more choice”. Faduma additionally related that:

“After I gave birth, …they cut you… and she [*her doctor*] did too far. …when she put the two pieces back together she didn’t do [it] the right way. So …I don’t like it… …maybe when you in labour they don’t know how to deal with it [FGC] so they just do …whatever. And it is you who is going to pay the consequences. It is very difficult. I do not like it.”

The comparison of women with FGC’s medical treatment with women from the country to which women with FGC have immigrated was also found in Manderson’s study of Sudanese women with FGC in Australia. There, women with FGC knew very well that Australian women had their episiotomies closed and vaginal openings tightened while physicians refused to close their vaginal opening [54].

In response to being asked to describe what their bodies felt like, the women questioned how they could describe what their bodies were like, since it was the only body they knew, not having another to compare it with. Faduma explained that her own body was all she knew, and her perspective could only be broadened by comparing bodies of women who have undergone FGC to women who have not. To further explore their own feelings of “normalcy”, participants situated themselves by referencing the experiences of other Somali women who had gone through FGC as well as Canadian women who had not gone through FGC. Xaawo described that in Canada, “everything you have to do for yourself,” whereas in Somalia, a woman who has gone through FGC receives “much more help and support.” Fawzia compared Somali to Canadian women in terms of differences in their sexual lives, suggesting that because of their FGC, Somali women have “a different feeling” during intercourse.

In the context of FGC not being a global norm, the women in our study wondered if they were ‘normal’. The differing norms of Somali and Canadian cultures highlight an underlying tension—a normal, Somali body was achieved for our participants by undergoing FGC. But, in Canada their bodies were deemed different and abnormal in comparison to the typical Western body. However, the women also realized that what was normal in Somalia, may not be so normal in Canada.

For women with FGC who have immigrated, not only are FGC’s outcomes being more critically evaluated by younger generations [55], but also its relation to religion is being critically questioned [55]. We heard this from a number of our participants who said they were taught that FGC was a religious practice, but upon arriving in the West and discovering that FGC was not necessarily a religious act, they felt misled. Faduma recalled:

“Growing up…we [*were*] told it was something to do with the religion…but at the end we found out it has nothing to do with religion…[*it*] was just a cultural, traditional… After I found out that I was so upset.”

Leylo compared her cultural group to other Muslim groups who do not practice FGC to highlight that it is not a religious practice:

“Some people think its religious… but it’s not…All the other Muslims are not circumcised except…Ethiopians and Somalis …but it has no religious background… it’s a culture.”

Hani similarly accounted that she would not have wanted to have FGC if she understood that it was not a religious practice and that other Muslim groups in comparison do not undergo it. Upon immigration to a country that does not practice FGC, the cultural value intrinsic to the practice becomes questioned [18].

Comparing themselves to other groups that practice FGC and finding that it was not religiously based troubled women’s identity, especially within the context of immigration. For our participants who were first generation, this seemed like a moment in time when normalcy of the past clashed with what was normal of the present and represented a moment of changing identity.

Overall, being normal was of importance to the participants in this study. When asked about how she felt in her body, Canab responded, “I am normal, normal.” Participants contemplated normalcy within their past and present contexts by comparing themselves to others within those respective communities.

### Theme 3: Feeling in my body

When participants talked about their bodies in their daily lives the picture of busy, productive women emerged. Women discussed their daily activities, what they expected of themselves, working hard, and when they felt their best. Overall, women were positive about their bodies and how their bodies felt in their lived lives. Women were also asked about bodily pain and pleasure in their daily lives. Even women who discussed pain in their lives ultimately expressed contentment.

#### Feeling

The prompts asking the participants to describe a day in their life, how their bodies felt within those lives, and when they felt best and worst, prompted the women to discuss their everyday work, both occupationally and domestically. Hani described the normalcy of working hard and how all women are tired. Despite leading tiring and busy lives, the women also reported feeling good in their bodies. Additionally, discussed was the importance of “feeling”, and a sense of loss when the women did not “feel”.

Participants' positioned themselves within the normal in describing how their bodies felt. They stated that the nature of women’s lives is busy, tiring, and marked by pains, especially with respect to work in and outside of the home. Leylo described “rushing with everything… always juggling ten things at a time”, including work and familial duties, struggling to relax. Jawaahir also described balancing caring for her children, running a household and working outside the home simultaneously. Some women described waking up as early as 5 am to pray and begin their occupational work before caring for their children. Overall, participants were clear that they worked hard, felt stressed, and led busy lives.

In spite of early rising and their busy lives, participants also reported feeling good in their bodies, finding them serviceable and enabling them to engage in day-to-day living. The women described feeling best in their bodies when they were able to relax on weekends and spend time with their families. Sahra said the weekend was when she felt best since she received help from her children with chores and was able to take the time to enjoy her family’s company. Faduma described the weekend as the time where she was able to do something for herself, such as make a nice breakfast, read and relax. Xaawo also described the weekend as the time when she felt the best, since this was when she was able to get a lot of rest.

When describing how they felt in their bodies, it became evident that “feeling” was important to the women. When they didn’t feel, it was expressed as a loss. Hani expressed her disappointment and loss of feeling because she had a c-section instead of being able to “feel” her labour by a vaginal birth. Sahra, on the other hand, experienced a loss of feeling during sexual intercourse as well as feeling pain. She indicated that she felt pain, but she did not feel either desire or pleasure:

“I’m not feeling well when I’m…[having] intercourse, and little bit feel…a pain…I didn’t feel even I need it [*intercourse*]…My husband [has] feeling, but I didn’t feel it.”

Although participants in our study expressed lack of feeling as a loss, the loss of their clitoris was not the focus; rather, it was feeling in general that was lost. In England [28] and Norway [18], women with FGC describe experiencing FGC, itself, as loss. The women in our study did not express this and conveyed a strong sense of being a whole being and not one with missing parts. It may be that women did not report this because we specifically did not focus on women’s genitals, or potentially, because in general, Canada has a climate of resettlement that is generally more accepting of Somali immigrants than some other countries [6]. Despite Canada’s potential strength of resettlement, there are other clear areas in need of improvement for immigrants, such as healthcare.

The word “feel” also referred to pleasure (“my husband [*has*] feeling”). Faduma talked about "feeling" during sexual intercourse but described that some women she knows did not “feel” anything. In contrast to the women she knows, Faduma said: “I do feel!” in reference to her experience with sexual intercourse. Canab also used the word “feel” to describe pleasure. She said, “When he puts his hands on me, I feel so much.”

#### Pain and pleasure

Pain was not mentioned until we asked about it directly. Pain in current life (including full-body achiness, sexual pain, back and neck pain) were also described in the context of being both provoked by daily activities or ongoing. Many of the participants described difficulty, or pain that they experienced and pushed through in order to fulfill their duties and obligations. Aamiina said that often she struggled to push herself to get out of bed in the morning because she feels pain. She said, “I feel pain… [all over] my…bone and body.” Faduma and Jawaahir described experiencing a different kind of pain in their necks and backs exacerbated by work outside the home. Xaawo similarly described feeling immense fatigue and frequent headaches.

Many attributed some pain, at some point in their lives, to being circumcised. Pain was discussed as something that had happened in the past, reverberating at the time of their circumcision, and also as resultant from having had FGC. Leylo described the pain of circumcision itself, especially in the context of urination after the procedure, although she experienced the procedure with anesthetic. Hani also discussed pain during urination when she first went through FGC, and also during menstruation. She described her pain from menstrual cramps as feeling “like labour”. Pain attributed to having had FGC was described by Leylo, who experienced difficulty of first having sexual intercourse because of her FGC, and further highlighted how having FGC affected her childbirth:

“I know circumcision was very hard when I get married…and when I was having my children… that’s when it hit me: ‘Oh, I was circumcised.’”

Xaawo also recounted the pain of childbirth, describing how the doctors “Cut you and then have to fix the cut”, exclaiming that “So much pain comes with it all!” Faduma reported feeling a sharp pain during sexual intercourse which made her desire to engage in intercourse decrease. Hani also accounted that after she was married and deinfibulated, her pain subsided to more “normal” levels.

Other studies report acute, physical pain: pain of first circumcision [56], pain during urination [57], pain during menstruation [44, 58], pain of deinfibulation [18], pain during first (or current) sexual intercourse [58, 59], and pain during childbirth [60]. Illustrative is a saying in Somalia that similarly describes the three pains or sorrows of women’s lives [61]: circumcision, deinfibulation and child birth. One interpretation of daily pain described by Somali women in Norway is that it is sociosomatic, an expression of the pain of loss and immigration [18]. It is interesting to speculate that the daily physical pain described by our participants might not be so widespread in Somalia. The experience of pain is context dependent [62, 63] and it is possible that the physical pain reported by our participants would be less in the context of a culture that validates FGC.

However, accepting pain as a part of women’s lives–even when it does not relate to female-specific biological events–is a pervasive cross-cultural reality. The International Association for the Study of Pain recognizes that insufficient attention is paid to women and pain [64]. The prevalent attitude that pain is a part of women’s lives, hinders a greater understanding of pain by researchers and clinicians. Furthermore, it prevents women from taking their pain seriously, being taken seriously by others (including healthcare providers) and thus accessing timely treatment.

Although participants talked about pain in their daily lives, they generally stayed away from labeling themselves as *in pain*. A member of the CAG explained to us that expressing pain is discouraged among children in Somalia. Others report that children are told to control themselves and are seen as strong for hiding complaints [65]. In this context, the label, "pain", is reserved for needing urgent help [66]. The CAG explained to us that the Somali word, *Xanuun*, the word used for pain, covers a broader set of connotations than the English word "pain". “*Xanuun*” includes pain, ache, sickness or illness, and something that affects your mobility and capacity [65]. The women in this study may have been responding to this wider meaning of “*Xanuun*” when they said they were not in pain. Regardless, they did not view themselves as in pain, or ill.

While women reported daily pains, variations of the phrase, “I feel fine” came up across most of the interviews. Despite Leylo’s descriptions of difficulty and pain, when asked how her body currently felt, she said she felt fine and healthy: “My body feels fine. I was fortunate that I was healthy.” Although Xaawo described the pain of childbirth due to her FGC, when asked how her body currently felt, she said she felt “fine” since she was “not sick”. In spite of Faduma’s description of pain during intercourse, when asked how her body felt, she said, “My body feels fine.” Even when Hani described her menstrual pain, she still said, “But this is normal and fine”. She went on to explain, “I just go about my day. I do not have to adapt because my body feels fine”.

Despite their aches and pains, and overall busy and tiring lives, the women are carrying on and this points to a resilience and getting on with life. It is possible that the word “fine” signifies acceptance of one’s suffering or pain that was ignited in the past and cannot be changed. The literature has used terms such as “stoic” [65] or enduring [66, 67] to describe habits of pain labeling. We feel reluctant to use such labeling because when asked to talk about “any place on their body” (and not just the genitals), the women in this study did say they had aches and pains within the context of their daily lives.

Women spoke directly about pleasure. Pleasure was also discussed in terms of ‘feeling good in my body’, intimacy with one’s partner, relaxation, and with respect to specific parts of the body. When asked if there was any particular place on their body where they experienced pleasure they talked about their breasts and necks. Similarly, Nigerian women with FGC report that their breasts are the most pleasurable and sensitive part of their body [68]. There was a pride and a strength in talking about pleasure, and it was an important part of the women’s narratives and explanations of being whole and well. It might be instructive to both our understanding of women with FGC and women’s sexuality in general to ask more questions about pleasure.

## Discussion

We studied Somali-Canadian women’s daily experience of their bodies in relation to their having had what they referred to as, “Pharaonic” circumcision and which was most likely Type IIIb FGC. We were interested in better understanding how FGC affected their current daily lives and potentially, longer lasting, whole body sensations. In line with interpretive phenomenology, we wanted to understand how our participants experienced the world through their bodies—bodies that had been affected by FGC. Our approach in the qualitative interviews was to ask both about their FGC experience and their sensations in their current daily lives. While we inquired about their FGC experience, participants were recruited with the understanding that this study was not about FGC per se but about their lived experiences in Toronto and, in particular, because part of the larger study is about pain specifically, pain and their brains (separate report). The fact that the focus of study moved from their genitals to their whole bodies—and in particular their daily experiences—may have relieved the women and taken a generally taboo topic and made it discussable. This suggests that what was in Somalia an undiscussed topic, in the current context was possible to discuss, perhaps because we were not interested in FGC as genital modification.

*Joyful women*: The atmosphere in our interviews was unusual. Women laughed at their follies, misunderstandings about what was to happen with respect to FGC, fears, and their resistance. They were also open to sharing their stories. Interviewers and interviewees shared this laughter and for the most part, the interviews conveyed women with the ability to have a meta view of their own experiences, a drive toward intimacy, irony, and a zest for life. Laughter was often used after stories of resistance as well as those of begging their mothers to let them have FGC suggesting that laughter might also cover discomfort with not doing what was expected of them but also conveying a sense of pride in their own independence and self-efficacy. There was also laughter in telling about their current lives. Thus, we got a strong sense of busy, committed women with a joy of life and eagerness to share their stories.

This is not always conveyed in other reports of Somali women who have immigrated to the west [56]. We may have perceived this in our participants as their education may have given them the skills to take a meta view on their circumstances; most women we interviewed had at least a high school education and a few had more than that. It might have been that most had been in Canada for at least 6 years and this might have given them a sense of comfort with non-Somalis that participants in other studies have not had. At least half came from families of relative means and from the city and this might have meant that in their natal countries, their families had some control over their lives. It may also be that having told the women that we were not interested in their genitals relieved them of the burden of talking about something culturally shameful. Whatever the reason, we think it is important to acknowledge that in spite of difficult circumstances, the pain of the past as well as the pain of their immigration, and potential continuing physical pain, these are strong, joyful, and resilient women.

*Immigration*: The daily lives of Somali-Canadian women cannot be considered outside of the context of their immigration. Yet, our participants did not address their immigration stories, explicitly. A few noted that they had already been abroad either for work or study, and they simply did not return home. One member of the CAG told us that they thought they would be returning to Somalia—they had no idea they would end up never returning. They also discussed some of their unpleasant experiences in the health care system in the context of their immigration. Berggren, Bergstrom and Edberg similarly describe that Somalian, Eritrean and Sudanese women report feeling different, especially in the context of traversing the Swedish healthcare system—especially, maternity care. They describe the “double shame” that women with FGC experience since it was shameful to not have FGC in their natal countries, but also shameful to have FGC in Sweden [56]. Immigration, itself, and being in a new country in which FGC is stigmatized is the context in which they currently wondered if they were normal since it is against the backdrop of a ‘new normal’ to which they compared themselves. However, based on their FGC stories, being normal was of paramount importance even before they immigrated to Canada. Beyond this, their immigration experience was not a focus for them. It is possible that discussing this would have been too painful and that we would have needed to prompt further. It is also possible that having lived an average of 10 years in Canada, they felt Canadian. Canada has the notion of a mosaic of cultures rather than a melting pot [69] which may have allowed our participants to feel a part of the place to which they had immigrated.

It is also possible that settlement practices in Canada relieved some of the stresses of immigration that women going to other countries might have experienced. In comparison to other diaspora locations such as London, England, Toronto at the time that many of our participants arrived had settlement strategies for Somali immigrants that seemed to help them integrate into their new communities as opposed to London, where Somali women characterized their daily experience as being one of ‘otherness’ and marginalization. Somali-Toronto women reported experiencing the same struggles of many immigrants including unemployment and culture clash, but without the same level of alienation [6]. The Scandinavian literature has also highlighted the difficulty that women with FGC experience, adjusting to a culture that is dissimilar from their own [67, 70] to which sociosomatic pain may have been a response [18].

Nevertheless, the insecurity and loss to Somalis who experienced the civil war cannot be underestimated, whether it was a loved one, their home, or even watching a country they loved become war-torn and unsafe [71]. Because of the Somali civil war many of our participants entered Canada as asylum seekers in the early 1990s and may have been subject to the hardship that many Somali immigrants faced becoming aquatinted with the very different social, economic, linguistic, religious and political climate within Canada [72]. Aside from political strife, Tiilikainen aptly points out that the Somali community had “lost its common trust and belief in the future” breeding a sense of “insecurity, hopelessness, and helplessness” ([71] pg. 64). We neither heard about this or saw it expressed in our participants. This may have been true for them at one point in time, but perhaps because most had lived in Canada since the early 1990s, it was less of an ever-present focus of their lives.

### Themes

Three themes emerged from our prompts: *Every Body Had It*: *Discussing FGC*, *I’m Normal Aren’t I*?, *and Feeling in My Body*.

#### Every body had it: Discussing FGC

It was important to us to understand women’s FGC stories since FGC happened in Somalia and we believed that this contributed to the bodily and social experiences the women had in Toronto. Women’s FGC stories conveyed resignation, but not bitterness, about their past: “What’s done is done; Something you have to pass.” They also conveyed the temporality of FGC: when it was to happen, the after effect, as a mark of culture that was carried forward into Canada—even with reverberating effects in later life activities. We did not ask about sexual feeling or child birth directly, although the women did speak of them in the context of their FGC.

In spite of conveying pride about what they have undergone for their culture, only one person, one who had been in Canada the shortest period of time, suggested that they were sorry their daughter could not experience FGC. All other participants expressed that FGC was not necessary in Canada and they did not see it as vital for their daughters. It is interesting to note that all our participants were first generation immigrants and middle aged and were mostly against continuing the practice of FGC with their daughters, suggesting that they were engaging with the Canadian culture and that their thinking had been changed within the time of their immigration.

The ways that different generations take up FGC can be dictated by how traditional the generation is, and thus how critical individuals are of the practice, especially in relation to religion. Generally, older generations, who tend to be more traditional, believe that there is a connection, even if it is a weak one, in the Hadith that promotes FGC [73]. Not only are older generations more likely to support FGC without being critical of it [55], but women who perceive themselves as being traditional have also been shown to be more likely to circumcise their daughters [73]. In Norway, Somali immigrants feel immense pressure from their traditional parents and grandparents to circumcise their girls [74]; not circumcising their children will bring shame on the family [66]. Although our participants did not reflect on how traditional they were, they did explicitly mention that they had determined that FGC is not mandated within Islam and would not have their daughters cut.

This suggests changing attitudes about FGC in the diaspora [74]. Changing attitudes, in turn, opens the question of how the mother/daughter dyad fares without this important bond between mother and daughter. Malian and Egyptian mothers with FGC report similarly not wanting FGC for their daughters in Canada but are concerned about finding new ways to uphold the values expressed by the practice—such as purity and virginity—without subjecting their daughters to the practice. To do this, mothers partake in explicit discussions with their daughters about relationships and sexuality [36]. Somali mothers in Finland report taking part in and also encouraging their daughters to wear a veil to display modesty and “preserve their culture and identity”, providing them with a “sense of security” ([71] pg. 62).

#### I’m normal aren’t I?

While strictly speaking, we were interested in the women’s current lives and how their bodies felt doing daily activities, we asked about their FGC story because we believed that how their bodies felt currently would be shaped by their early life FGC experiences. Perhaps because of the juxtaposition of their lives in Somalia with their lives in Canada, a common thread of wondering what was normal ran through their FGC story and into their current lives. Women made comparisons of their earlier culture to the Canadian one and to others in the Canadian culture. They conveyed a strong sense of wanting to be normal and they grappled with what was normal in both the Somali and the Canadian context. For a number, finding out that FGC was not religious and, “just culture”, was a jolt to their sense of the importance and power of their having had FGC.

Within Somalia, FGC made a normal body and improved prospects for the future. As Boddy says of the Sudanese women of the village she called, “call Hofriyat”:

“For them, the practice of FGC was *normal*, taken for granted, seldom even discussed among themselves. Though acknowledged to be very painful, the experience was looked forward to, while the prospect of not being cut was horrifying.” ([75] pg. 41).

As she points out, “…the social order is internalized…and power is embodied, made self” ([75] pg. 50). Imagine then, the transference of that body, that self and its concomitant internalization of social powers, to Canada, where FGC makes an abnormal body. Thus, a deep irony emerged from this juxtaposition; while women overwhelmingly talked of having had FGC to be normal, having immigrated to a country that does not practice FGC, they wondered or “realized” they were not considered normal. Even as late as 10 years post-immigration, women were grappling with this, both asserting they were normal and asking if they were normal.

This may suggest a continuing struggle with identity. In other studies, participants used the word, “normal”, in reference to delivery and labour [76], sexual intercourse [19, 44, 77], deinfibulation [77], and reinfibulation [19]. Despite this, there is little reporting relaying women’s self-reflection of their own normalcy. Grappling with two very different definitions of normalcy and trying to reconcile both, despite their disparities, was an important point of reflection for the women in our study.

Of course, what is normal and not normal consumes many women’s thinking. Many women reflect on their own vulvas as “abnormal”, leading them to desire Female Genital Cosmetic Surgeries [75, 78]. There is much work, generally, on women as the bearers of normal culture. As is the case for Somali women, ‘gendered role prescriptions’ are passed on from mothers to their daughters. Mothers ensure that their daughters look and behave according to the norm in order to be accepted into society [79]. Daughters, across cultures, are often expected to take up these cultural roles that they learn from their mothers, to derive social status [79]. Consistent with this, the role of Somali women is to create a body for their daughters that would be normal and therefore, acceptable in society, allowing a promising future [19, 33]. In the case of our participants, the juxtaposition of what was normal in Somalia with what is normal in Canada suggested a heartfelt struggle with their identity and trying to put the two pieces of their lives together.

#### Feeling in my body

In spite of FGC being determined by mothers, grandmothers, and the society at large [33] our participants expressed ownership of their bodies. A number even took ownership of FGC seeing themselves as demanding and pushing for it. The women in our study did not describe their bodies as missing something—rather, they described their bodies as capable and full. However, they were concerned about feeling. Some described not feeling or feeling different than their husbands during sex. Some conveyed a sense of loss—but it was loss of feeling and not of a body part.

The women realized that there were many different bodies and without direct comparison, there was no way of knowing how theirs was similar or different from another person’s. They spoke of it as passing through time with them—“the only body I’ve ever known”—and as serviceable in their daily lives. They acknowledged aches and pains, including the difficulties of meeting all of life’s demands, but they welcomed rest and relaxation as when their bodies felt best.

Discussion of bodily sensations was situated in embodied life experiences, including negative healthcare experiences. Women worked at normalizing their bodies in a culture that views them as different. If there was pain, it was pain all women feel. If there were aches, it was just a normal part of life.

Throughout the narratives of normalization were also indications of bodily pain including but also beyond the vulvar region. It may be that the aches and pains of daily life that our participants reported are also the sociosomatic pain described by Somali-Norwegian women [18]. However, it is also possible that our participants had continuing neuropathic pain from the FGC [25]. Pain in multiple body sites [80] as well as excessive tiredness [81] are all signs of chronic pain which warrants further study. The physicality of pain in women with FGC is not well understood as of yet is because participants do not report it explicitly, viewing pain as a normal part of life. Culture is a significant factor in how an individual reacts to pain [63]. In Somali, the literal meaning of pain is ‘sick’ and our participants did not consider themselves ill.

Chronic pain conditions are disproportionately represented in women and racial minorities [82, 83, 84]. Importantly, people with visible minority status are at a higher risk of under treatment of their pain due to their own beliefs about the pain experience and the ability of healthcare practitioners to communicate adequately with them [85, 86]. This has been found to be the case for Somali women giving birth in Sweden [87] and the United States [13]. In general, there is little knowledge of cultural beliefs and preferences on the experience of pain [88]. The International Society for the Study of Pain recognizes that: “Pain conditions affecting women have a significant global impact. Yet, there is still a lack of awareness/recognition of pain issues affecting women” [89]. Since many cultures believe that pain is a normal part of women’s lives, including western cultures, women with chronic pain conditions do not often receive the care they need and Somali women are no exception.

### Strengths and weaknesses

A strength of this study is that while we asked women with FGC about their current lives, we did not isolate their lives as being only about FGC or only about immigration. We studied a group of women with FGC in a relatively understudied area (Toronto, Canada).

The group of Somali women we studied were recruited by purposive sampling and were known to members of our community advisory group (CAG) who did the recruiting. Although they were not a homogeneous group in themselves, they may have shared characteristics because they were known to our CAG. Some may have been willing to participate in our study because of their outgoing personalities. As well, many of them immigrated to Canada in the 1990s and had been settled in Canada for a long time (average of 14 years), differentiating them from other diaspora groups of women with FGC being studied [90]. Because of this, our results may not be generalizable to newly arrived Somali immigrant populations, or other populations with FGC in a general Western context. Previous literature also often incorporates women with FGC from many African countries including Somalia, Sudan and Ethiopia, all with different micro-cultures [43]. Differently, the current study focuses only on Somali women. Thus, our findings should be viewed as specific to Toronto-Somali immigrant women. However, having found some characteristics not described in other studies, it might be worth considering them in future studies.

While Somali interpreters were present at the interviews and transcripts that were in Somali were translated into English by a Somali speaker, language nuances always exist. For example, as discussed, cultural interpretations of pain and the Somali word for pain, Xanuun, are linked to sickness, weakness, etc., making the word, ‘pain’ as we know it, linked to being incapacitated and not just a sensation. The word, ‘feel’ was also used to denote pain as well as pleasure. We navigated this by taking words to the CAG for their help in collectively unpacking and defining language about pain and pleasure. That process spoke volumes on the power of perception of pain and pleasure being contingent on cultural understanding and expression.

## Conclusion

We asked Somali-Canadian women with FGC about whole bodily sensations—including pain and pleasure—in the context of their FGC and lived lives in Toronto. The interviews introduced busy and active women engaging in rich family, work and social lives. With few exceptions, our participants were vivacious and conveyed empowerment and strength. During the interviews, the tone did not turn somber, even when the women were discussing pain or feelings of betrayal. There were no protracted silences and laughter was frequent. The memory of FGC was strong and vivid but it was expressed with acceptance and resignation–as something that just is; that was normal; and that was done with much consideration by their mothers. Being normal was important to them and comparisons of themselves to their friends in Somalia as well as Canadian women was a strong theme. When asked about how their bodies felt, participants were concerned with understanding the context of what is ‘normal’, working hard to support their families in all aspects of their lives, considering themselves healthy in spite of having symptoms that might suggest chronic pain. Most women recounted pain and discomfort in their adult lives but were intent on not noticing or giving it power. The women generally considered that the life of a woman is filled with pain, especially reverberating at events that echo the original circumcision [65]. However, they did not view themselves as ‘in pain.’ In spite of difficulties, they dealt with both pain and pleasure in the context of their busy, daily lives suggesting remarkable resilience.
